# Plasma angiopoietin-2 is persistently elevated after non-small cell lung cancer surgery and stimulates angiogenesis in vitro

**DOI:** 10.1097/MD.0000000000004493

**Published:** 2016-08-12

**Authors:** Leng Zhou, Haidan Lan, Qinghua Zhou, Jianming Yue, Bin Liu

**Affiliations:** a Department of Anesthesiology; b Department of the Lung Cancer Center, West China Hospital of Sichuan University, Chengdu, Sichuan, China.

**Keywords:** angiogenesis, angiopoietin-2, anti-angiogenic therapy, non-small cell lung cancer, postoperative adjuvant setting

## Abstract

Angiopoietin-2 (Ang2) is a key proangiogenic factor, but its role in surgery-induced angiogenesis, a possible cause of cancer recurrence, is still elusive.

We measured the plasma Ang2 levels in healthy controls (n = 42) and stage I–IV perioperative nonsmall cell lung cancer (NSCLC) patients (n = 227) with enzyme-linked immunosorbent assay, and examined the impact of Ang2 in the plasmas on in vitro angiogenesis and proliferation of human umbilical vein endothelial cells and human microvascular endothelial cells.

Ang2 plasma levels are significantly increased in untreated NSCLC patients (2697 ± 1354 pg/mL) compared to control (1473 ± 560.6 pg/mL) and positively associated with disease stage but not with histology. Ang2 plasma levels in stage I–IIIA NSCLC patients (n = 154) are elevated after the standard open thoracic surgery, following an approximate pattern to increase quickly in the 1st postoperative days (PODs, from preoperative 2342 ± 1084 to POD1: 4485 ± 1617 and POD3: 5370 ± 1879 pg/mL), reach the peak about 2 weeks later (POD14: 6099 ± 2280 pg/mL), drop slowly thereafter (POD28: 3877 ± 1388 and POD42: 3365 ± 1189 pg/mL), and remain significantly higher than preoperative 8 weeks after the procedure (POD56: 2937 ± 943.3 pg/mL). The postoperative plasmas enhance in vitro angiogenesis and Ang2 removal from the plasmas can counteract the effect. The postoperative plasmas stimulate endothelial proliferation independently of Ang2.

These results suggest that plasma Ang2 increases after NSCLC surgery and contributes to the proangiogenic property of the postoperative plasmas, thus supporting the possible administration of anti-Ang2 therapy for NSCLC in postoperative adjuvant setting.

## 1 Introduction

Surgery remains the major treatment choice for nonsmall cell lung cancer (NSCLC). The 5-year survival rate of resected NSCLC, however, ranges from only 25% to 73% depending on the pathological stage of the disease. Local or distant recurrence is very common to the patients. ^[1]^

Among the many factors that may contribute to the high rate of cancer recurrence is surgery-induced angiogenesis, the formation of new blood vessels from preexisting vasculature. Surgical procedure triggers the release of angiogenic factors to local tissue and circulation. The resulting angiogenesis not only promotes surgical trauma healing, but also facilitates the survival and growth of residual cancer cells and remote micrometastases. [^2^ ^3^ ^4^] Indeed, plasma level and wound fluid concentration of proangiogenic factors, such as vascular endothelial growth factors (VEGF) and angiopoietin-2 (Ang2), have been shown to be increased after surgery for a variety of cancers.[ ^2^ ^4^ ^5^ ^6^ ^7^ ^8^] Postoperative plasmas from patients undergoing minimally invasive colorectal resection stimulated endothelial cell migration, invasion, and tube formation in vitro. ^[9]^ In murine breast cancer model, primary tumor removal led to upregulation of angiogenesis-associated genes and increase in lung metastatic burden. ^[10]^ In line with these findings, clinical data suggested that surgery-induced angiogenesis accounted for the early postoperative relapses of breast cancer. ^[11]^

Given the above observations, it is reasonable to incorporate the antiangiogenic tumor therapy in the postoperative adjuvant setting. [^2^ ^3^ ^4^] In fact, both preclinical and clinical studies have suggested the effectiveness of this strategy, even though the studies were not particularly designed to target the surgery-induced proangiogenic factors. Srivastava et al ^[12]^ demonstrated that anti-Ang2 antibody treatment suppressed angiogenesis in metastatic nodules and blocked metastases of breast cancer and Lewis lung carcinoma in mouse models of postsurgical therapy. The combination of anti-Ang2 with metronomic chemotherapy further decreased the metastases and increased overall survival. Administration of angiogenesis inhibitor tetrathiomolybdate beginning 4 to 6 weeks after surgery doubled the progression-free survival time for stage I and II malignant pleural mesothelioma patients as compared to the nontreated patients. ^[13]^ The postsurgical antiangiogenic therapy should be more effective without affecting wound healing processes if additionally the surgery-induced proangiogenic factors can be timely neutralized based on their plasma dynamics. ^[3]^ On the other hand, however, phase 3 trials showed that VEGF inhibitory antibody bevacizumab failed to improve postoperative disease-free survival or overall survival for colorectal cancer and triple negative breast cancer patients, [^14^ ^15^ ^16^] implying that VEGF pathway inhibition in the surgical adjuvant setting may not provide more benefits for patients, and that finding and using other molecular targets may be necessary. ^[12]^

Ang2 was proposed as a potential target for antiangiogenic drug development.[ ^12^ ^17^] Normally produced by endothelial cells and functioning as a ligand for Tie2 receptor tyrosine kinase, Ang2 is preferentially expressed in both tumor endothelial and epithelial cells in a variety of cancers, including NSCLC, and promotes tumor angiogenesis by priming the vasculature. ^[18]^ Patients with NSCLC have higher plasma Ang2 level, and that is further elevated for at least 30 days after tumor resection.[ ^6^ ^7^ ^19^] The changes after 30 days, especially in the 2nd month, a critical time for starting postoperative adjuvant therapy, ^[20]^ however, is unknown. Unknown also is the impact of postoperative plasma Ang2 on angiogenesis. The major purposes of the present study were to examine patient plasma Ang2 level change during the 1st 8 weeks after the standard open thoracic surgery for NSCLC, and to assess the effect of Ang2 in the postoperative plasmas on endothelial cell tube formation in vitro, thus providing evidence to evaluate the appropriateness of using anti-Ang2 cancer therapy in the postoperative adjuvant setting.

## 2 Methods

### 2.1 Study population, blood sampling, and processing

Forty-two healthy volunteers (control, 27 male, 15 female, age 55.2 ± 8.7) and 253 NSCLC TNM stage I–IV patients were enrolled into the study, and all the controls and 227 patients (166 male, 61 female, age 57.9 ± 10.6) completed the study. The patients were treated in the Lung Cancer Center, West China Hospital of Sichuan University, China, in the year 2014 and 2015. Three to 5 peripheral blood samples, 2 to 4 mL each, were taken from each stage I–IIIA NSCLC patient undergoing standard open thoracic surgery (including wedge resection, segmental resection, sleeve resection, lobectomy, or pneumonectomy) without any preoperative therapy: 1 immediately prior to surgery (POD-1), and others at postoperative day 1 (POD1), 3 (POD3), 10–17 (POD14), 25–32 (POD28), 37–47 (POD42), and 51–61 (POD56). Samples from patients experiencing postoperative adult respiratory distress syndrome or infection that were known to associate with increasing plasma Ang2 level were removed from this study. One blood sample was taken from each control, and each stage IIIB–IV NSCLC patients before any treatment. Blood samples were collected in EDTA-containing tubes and centrifuged at 1000 *g* for 10 minutes. Plasmas were isolated and stored at –80 °C until analysis. The study protocol was approved by the Ethical Committee of Sichuan University. All patients and volunteers signed a written informed consent before entering the study.

### 2.2 Ang2 measurement

Plasma Ang2 concentration was determined using Ang2 human enzyme-linked immunosorbent assay kit (ab99971, Abcam, Cambridge, MA) according to manufacturer's instructions. Briefly, standard Ang2 and 1:5 diluted plasma samples were pipetted into wells on 96-well plates coated with specific antihuman Ang2 antibody. The wells were washed and biotinylated antihuman Ang2 was added, followed by HRP-conjugated streptavidin and then substrate TMB. Intensity of the reaction color was measured at 450 nm. All measurements were performed in duplicate. Experiments were repeated twice.

### 2.3 Cell culture

Human umbilical vein endothelial cells (HUVECs, CC-2519, Lonza, Walkersville, MD) were grown in endothelial growth medium (EGM) Bullet Kit (CC-3124, Lonza). Human microvascular endothelial cells (HMVECs, CC-2543, Lonza) were grown in EGM-2MV Bullet Kit (CC-3202, Lonza). Cells are maintained at 37 °C in a humidified 95% air and 5% CO_2_ atmosphere, and were used within 8 passages.

### 2.4 In vitro angiogenesis assay

Ninety-six-well plates were coated with 50 μL/well growth factor-reduced Matrigel (Corning Life Sciences, Tewksbury, MA) and incubated for 1 hour at 37 °C for gel solidification. After starvation in endothelial basal medium (CC-3121, Lonza) supplemented with 0.1% (w/v) bovine plasma albumin for 16 hours, HUVECs or HMVECs were trypsinized, resuspended in diluted plasmas, and seeded at 20,000 cells/well. Pooled plasmas, with or without Ang2 removal, from either healthy controls or patients were diluted 1:3 with EGM. To remove Ang2, the plasmas were treated with Ang2 specific antibody (sc-74403, Santa Cruz Biotechnology, Dallas, TX) conjugated M-270 Epoxy beads prepared using Dyna beads Antibody Coupling Kit (14311D, Life Technologies, Carlsbad, CA). Concentrations of the pooled samples, with or without Ang2 removal, were measured with enzyme-linked immunosorbent assay. Images (50× magnification) of tubular network were taken with a Leica Microsystems 4000B microscope equipped with a RTKE Spot camera (Diagnostic Instruments, Sterling Heights, MI) 12 hours after cell plating. Tube branching points presented by the image were counted with ImageJ (National Institutes of Health, Bethesda, MD). Experiments were performed in quadruplicate and repeated twice.

### 2.5 WST proliferation assay

HUVECs or HMVECs were starved as for in vitro angiogenesis assay. Cells were trypsinized, resuspended in EGM (1:2 diluted with endothelial basal medium), and seeded at 3000 cells/80 μL/well to 96-well plates. Pooled plasmas (40 μL) from either healthy controls or patients, with or without Ang2 removal, were added to each well beforehand. Cell proliferation was determined 12 or 72 hours later according to the manufacturer's instructions. Briefly, 13 μL of WST-1 (Roche Diagnostics, Indianapolis, IN) was added to each well, followed by a 1-hour incubation at 37 °C. Absorbance at 450 nm was measured using POLAR star Omega microplate reader (BMG LABTECH, Ortenberg, Germany). Experiments were performed in quadruplicate and repeated twice.

### 2.6 Statistical analysis

Data were demonstrated as mean ± standard deviation. For plasma Ang2 level, the Mann–Whitney *U* test was used to compare patients and control groups, and the Kruskal–Wallis test was used to compare several groups. The Ang2 levels at different time points within a group were compared using Wilcoxon matched pairs test. These tests were chosen because the values did not fit normal distribution. For in vitro angiogenesis branching points, one-way ANOVA test was used to compare several stimulation experiment groups, while the Student *t* test was used to compare the paired inhibition experiment groups. All statistical analyses were performed using SPSS version 19.0 software (SPSS Inc., Chicago, IL). Results were considered statistically significant for *P* *<* 0.05.

## 3 Results

### 3.1 Ang2 plasma levels are increased in untreated NSCLC patients and associated with disease stage but not with histology

**Figure 1:**
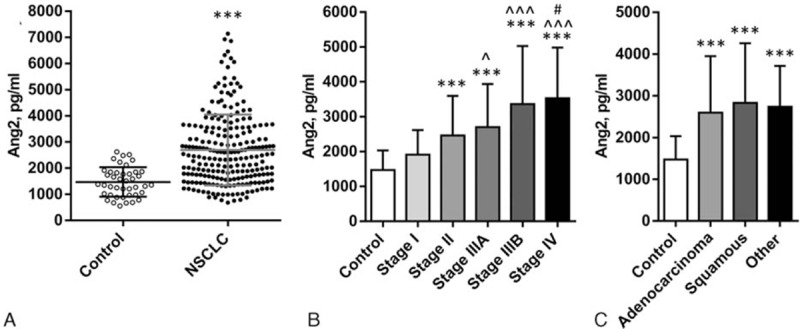
Ang2 plasma levels in untreated NSCLC patients and healthy control. (A) Overall, (B) by disease stage, and (C) by disease histology. Values are expressed as mean ± SD. ^∗∗∗^ *P* < 0.001, compared to control; ^^^ *P* < 0.001, ^ *P* = 0.03, compared to stage I; and ^#^ *P* = 0.03, compared to stage II. Ang2 = angiopoietin-2, NSCLC = nonsmall cell lung cancer, SD = standard deviation.

Patient clinical characteristics are presented in Table 1. We measured plasma Ang2 levels in untreated NSCLC patients (n = 227) and healthy volunteers (n = 42). Overall patients have higher Ang2 than controls (2697 ± 1354 vs 1473 ± 560.6 pg/mL, *P* < 0.001, Fig. 1A). With stage progression, plasma Ang2 increases significantly from the control basal level and stage I (n = 55) 1914 ± 705.2 (*P* = 0.054, compared to control), to 2464 ± 1132 (stage II, n = 51, *P* < 0.001), 2703 ± 1236 (stage IIIA, n = 48, *P* < 0.001), 3361 ± 1669 (stage IIIB, n = 37, *P* < 0.001), and 3532 ± 1449 pg/mL (stage IV, n = 36, *P* < 0.001) (Fig. 1B). All histologic types of NSCLC have higher plasma Ang2 than control, with 2596 ± 1353 in adenocarcinoma (n = 122, *P* < 0.001), 2831 ± 1426 in squamous cell carcinoma (n = 86, *P* < 0.001), and 2739 ± 977.8 pg/mL in other types (n = 19, adenosquamous 8, large cell 6, carcinoid tumor 2, unclassified 3, *P* < 0.001) (Fig. 1C). No significant differences in plasma Ang2 level were detected among the histologic types. Results from the normal population and untreated patients will serve as baseline references for postoperative data analysis in this study.

**Table 1:**
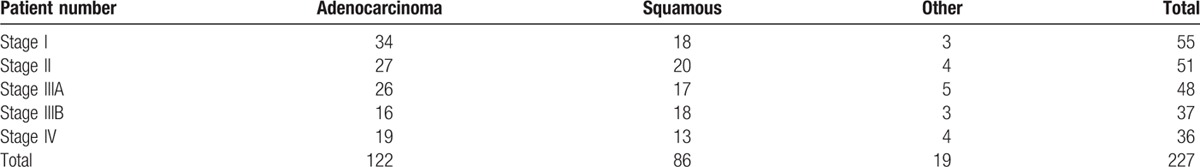
Clinical characteristics of nonsmall cell lung cancer patients.

### 3.2 Ang2 plasma levels in NSCLC patients are elevated after surgical removal of the tumors

Totally 154 patients (55 stage I, 51 stage II, and 48 stage IIIA) completed this study. All the patients had blood samples taken on POD-1, POD1, and POD3, and contributed 2 more late specimens on POD14 (actual day 10–17 after surgery), POD28 (day 25–32), POD42 (day 37–47), or POD56 (day 51–61). The late specimens were taken within the indicated period and were bundled to specific POD dates to permit statistical analysis. With patients’ cooperation, we managed to get 76, 79, 78, and 73 samples for the last 4 dates, respectively, and to collect the samples in a way to allow cancer stages and histologies distribute similarly among the groups to minimize their possible influence on the analysis.

**Figure 2:**
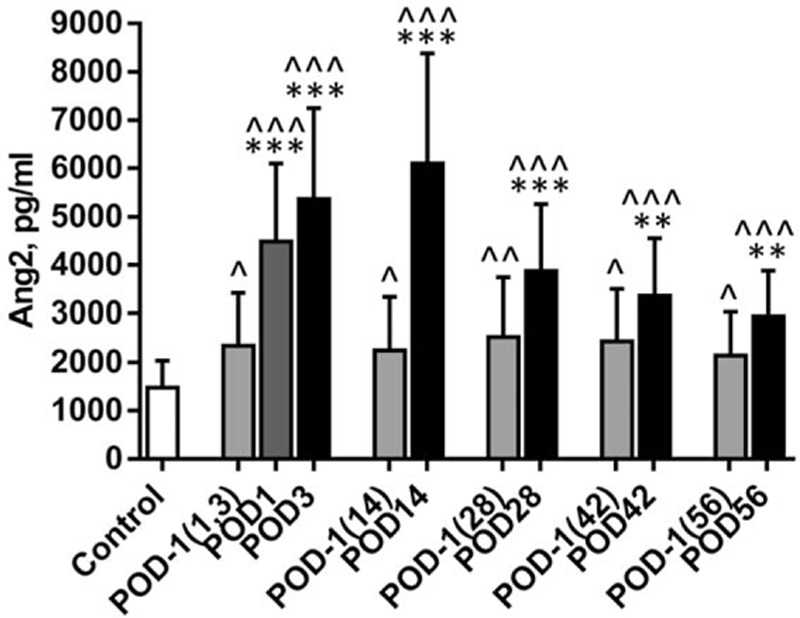
Ang2 plasma levels in perioperative NSCLC patients. Values are expressed as mean ± SD. ^∗∗∗^ *P* < 0.001, ^∗∗^ *P* = 0.003 (POD42) or 0.004 (POD56), compared to corresponding POD-1; ^^^*P* < 0.001, ^^*P* = 0.008, ^*P* = 0.02 (POD-1 (1, 3)), 0.03 (POD-1 (14)), 0.02 (POD-1 (42)), 0.04 (POD-1 (56)), compared to control. Ang2 = angiopoietin-2, NSCLC = nonsmall cell lung cancer, POD = postoperative day, POD-1 (1, 3) = preoperative control for POD1 and POD3 samples, POD-1 (14) = preoperative control for POD14 sample, POD-1 (28) = preoperative control for POD28 sample, POD-1 (42) = preoperative control for POD42 sample, POD-1 (56) = preoperative control for POD56 sample, SD = standard deviation.

We pooled the plasma Ang2 values from the same time point together, regardless of disease histology (based on the above results) and stage, and compared the means of the values with those of their own POD-1. Of note, Ang2 levels are elevated significantly at all the 6 time points in the 1st 8 weeks after surgery. Figure 2 shows the trend that Ang2 level increases quickly in the 1st days after the resection (from preoperative 2342 ± 1084 to POD1: 4485 ± 1617, and POD3: 5370 ± 1879 pg/mL, *P* < 0.001), reaches the peak about 2 weeks later (POD14: 6099 ± 2280 pg/mL, *P* < 0.001), and drops slowly thereafter (POD28: 3877 ± 1388, *P* < 0.001, and POD 42: 3365 ± 1189 pg/mL, *P* = 0.003), but is still significantly higher than its own preoperative value 8 weeks after the procedure (POD56: 2937 ± 943.3 vs preoperative control for POD56 sample (POD-1 (56)): 2140 ± 891.7 pg/mL, *P* = 0.004). Not surprizingly, all groups of patient plasmas, both of pre- and postsurgery, exhibit significantly higher Ang2 levels than that of the healthy control (Fig. 2). We further stratified the data according to disease stages. Consistently, the changes of plasma Ang2 levels show a similar pattern in all the individual stage groups (Table 2) to that in the pooled group (Fig. 2).

**Table 2:**
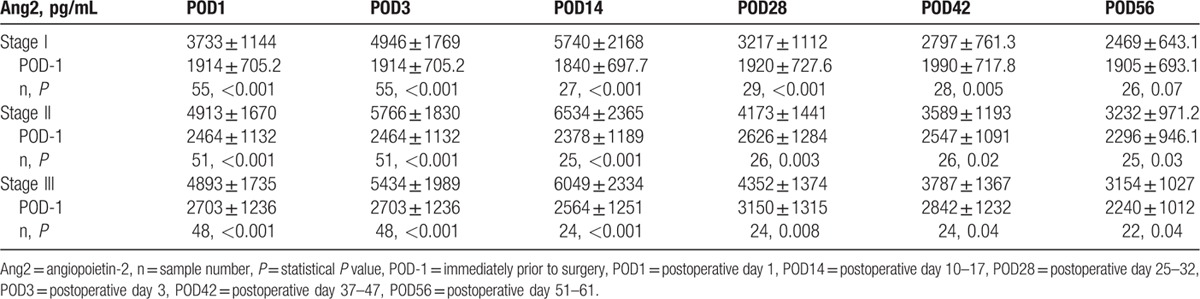
Plasma angiopoietin-2 levels in different stages of nonsmall cell lung cancer.

### 3.3 Postoperative plasmas enhance in vitro angiogenesis and the effect can be inhibited by Ang2 removal

**Figure 3:**
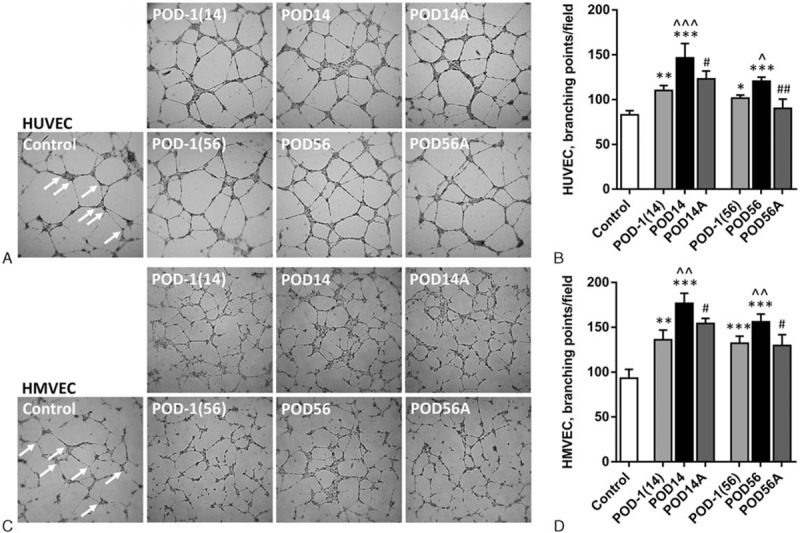
Effects of perioperative plasmas with or without angiopoietin-2 on in vitro angiogenesis. (A, C) Images of tubular network of HUVEC (A) or HMVEC (C); magnification: 50×; arrows: representative branching points; (B, D) quantification of branching points in 50× field for HUVEC (B) or HMVEC (D). Values are expressed as mean ± SD. ^∗∗∗^ *P* < 0.001, ^∗∗^ *P* < 0.008, ^∗^ *P* = 0.04, compared to healthy control; ^^^*P* < 0.001, ^^*P* < 0.007, ^*P* = 0.03, compared to corresponding POD-1; ^##^ *P* = 0.009, ^#^ *P* < 0.04, compared to corresponding nonantibody neutralized plasma. HMVEC = human microvascular endothelial cell, HUVEC = human umbilical vein endothelial cell, POD = postoperative day, POD-1 (14) = preoperative control for POD14 sample, POD-1 (56) = preoperative control for POD56 sample, POD14A = antibody beads treated POD14 sample, POD56A = antibody beads treated POD56 sample.

We combined 5 plasma samples that have the highest Ang2 concentration from each of the healthy control, preoperative control for POD14 sample (POD-1 (14)), POD14, POD-1 (56), and POD56 groups. Half of the combined POD14 and POD56 plasmas were treated with Ang2 specific antibody beads to remove Ang2. ^[21]^ Ang2 concentration of the resulting samples were measured to be 2322 (control), 4957 (POD-1 (14)), 11652 (POD14), 4260 (POD-1 (56)), 5264 (POD56), <100 (antibody beads treated POD14), and <100 (antibody beads treated POD56) pg/mL. These plasmas were tested for their effects on in vitro angiogenesis of HUVEC and HMVEC. These cells were chosen because that the early passages of the cells maintain most features of native vascular endothelial cells and that the cells have been shown to be valuable in vitro models to study the regulation of angiogenesis. ^[22]^ Besides, both HUVEC and HMVEC express Ang2 receptor Tie2.[ ^23^ ^24^] Compared to control plasma (branching points/field: 83 ± 4.8), all the patient plasmas exerted significant increase in HUVEC tube formation (Fig. 3A) as quantitated by the number of branching points (Fig. 3B). Both postoperative plasmas showed more potent effect than their own preoperative plasmas (POD14 vs POD-1 (14): 146.5 ± 16.1 vs 110.3 ± 5.6, 32.8% increase, *P* < 0.001; POD56 vs POD-1 (56): 120.5 ± 4.7 vs 101.8 ± 3.4, 18.4% increase, *P* = 0.03), and POD14 was the most prominent. However, when the Ang2 is removed, both POD14 and POD56 lost some of their power (antibody beads treated POD14: 123 ± 9, 15.4% decrease, *P* = 0.04; antibody beads treated POD56: 90.3 ± 10.3, 25.1% decrease, *P* = 0.009). HMVEC tube formation demonstrated a very similar response to the plasmas (Fig. 3C).

### 3.4 Postoperative plasmas enhance endothelial cell proliferation independently of Ang2

**Figure 4:**
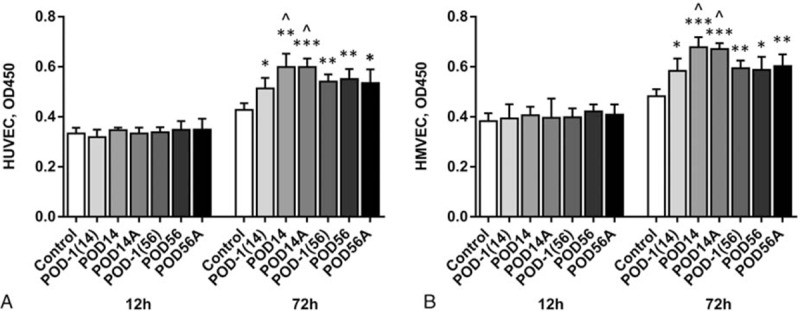
Effects of perioperative plasmas with or without angiopoietin-2 on endothelial proliferation. (A) HUVEC, (B) HMVEC. Cells were treated with plasmas for 12 or 72 hours. Values are OD450 and expressed as mean ± SD. ^∗∗∗^ *P* < 0.001, ^∗∗^ *P* < 0.007, ^∗^ *P* = 0.02, compared to healthy control; ^*P* < 0.05, compared to corresponding POD-1. HMVEC = human microvascular endothelial cell, HUVEC = human umbilical vein endothelial cell, POD = postoperative day, POD-1 (14) = preoperative control for POD14 sample, POD-1 (56) = preoperative control for POD56 sample, POD14A = antibody beads treated POD14 sample, POD56A = antibody beads treated POD56 sample, SD = standard deviation.

The increased in vitro angiogenesis may be confounded by the effect on cell proliferation exerted by the plasmas. To clarify this, we treated both HUVECs and HMVECs with the plasmas used for in vitro angiogenesis assay for 12 or 72 hours, and evaluated cell proliferation. As shown in Fig. 4, at 72 hours, both preoperative and postoperative plasmas from patients were observed to increase cell growth as compared to healthy control. POD14, but not POD56 plasma, enhanced proliferation compared to their own preoperative plasmas. Removal of Ang2 failed to deplete the plasmas’ capacity to stimulate proliferation. No plasmas showed prominent effect on proliferation when the treatment lasted for only 12 hours, the duration of in vitro angiogenesis assay. These data suggested that the postoperative plasmas can stimulate proliferation at certain time, but the effect is independent of Ang2.

## 4 Discussion

By measuring plasma Ang2 levels and evaluating its effects on endothelial cell tube formation and proliferation, we demonstrated here that Ang2 plasma levels are higher in untreated NSCLC patients than in healthy volunteers and associated with tumor stage but not with histology; that Ang2 plasma levels in patients are elevated after NSCLC surgery, with the approximate pattern to increase in the first PODs, reach the maximum 2 weeks later, drop slowly then, and keep higher than the preoperative value at least 8 weeks after the surgery; that the postoperative plasmas stimulate angiogenesis in vitro and the effect can be inhibited by Ang2 removal; and that the postoperative plasmas promote endothelial proliferation and the effect is independent of Ang2.

Ang2 is a strong proangiogenic factor in the presence of VEGF and has been associated with a variety of cancers.[ ^18^ ^25^] A few small-scale studies have reported that Ang2 plasma/serum levels are increased in NSCLC patients and associated Ang2 with the progression of the disease.[ ^6^ ^19^ ^26^ ^27^ ^28^ ^29^ ^30^] Our study confirmed these observations with a larger sample size and different geographic patient population (China mainland), helping to generalize these findings. The consistency of the baseline values with the results of other studies suggests the validity of our postoperative data.

Anti-Ang2 strategy was suggested as a potential 2nd-generation antiangiogenic cancer therapy, in view of the recognized limitations of anti-VEGF therapy.[ ^12^ ^31^] Actually, this kind of therapeutics has been actively tested in different clinical settings.[ ^17^ ^31^] Application of anti-Ang2 therapy in the postoperative adjuvant setting in preclinical models was shown to be promising. ^[12]^ The translation of these findings from bench to bedside, however, needs some prerequisite clinical data, among them is the perioperative dynamics of patient plasma Ang2. Available data from NSCLC showed that plasma/serum Ang2 is elevated at POD1, POD3, POD7, and POD30 compared to the preoperative levels.[ ^6^ ^7^] Based on the clinical practice that adjuvant therapy usually starts 4 to 6 weeks after surgery and that the bevacizumab therapy is recommended to start 4 to 8 weeks after surgery, ^[20]^ we extended the plasma sampling to 8 weeks after surgery. Our results revealed that Ang2 levels are sustained significantly higher from POD28 to POD56 than the preoperative and the healthy control. Supportive to this finding is the increase of Ang2 plasma level in the 2nd month after minimally invasive colorectal resection reported by Kumara et al. ^[9]^ Considering the Ang2 values in POD56 (2937 ± 943.3 pg/mL), preoperative (2140 ± 891.7 pg/mL), and healthy control (1473 ± 560.6 pg/mL), it is very likely that the plasma Ang2 will be continued high for some time 2 months after surgery.

Angiogenesis starts immediately after tissue injury and can last for 10 weeks, even throughout the wound healing process, including the remodeling stage. [^32^ ^33^ ^34^] Prolonged angiogenesis occurs in delayed wound healing, a common surgical complication. ^[35]^ Ang2 may be one of the driving factors for the angiogenesis. In fact, Ang2 was detected to be overexpressed in fibroblasts, myofibroblasts, and endothelial cells in healing human wounds for at least 52 weeks. ^[36]^ These may be some reasons for the lingering high level of Ang2 in the postoperative patients. In some cases, the possible growth of residual tumors and remote micrometastases may also initiate angiogenesis by releasing Ang2. ^[18]^ The long duration of high level plasma Ang2 caused by surgery may justify the administration of anti-Ang2 therapy for NSCLC in the postoperative adjuvant setting.

Our functional analysis showed that postoperative plasmas are stronger than preoperative plasmas in stimulating in vitro angiogenesis, whereas both are more potent than the healthy control. This is consistent with the reported observations about minimally invasive colorectal resection postoperative plasmas. ^[9]^ There are many elevated proangiogenic factors in the postoperative plasmas, such as VEGF, ^[9]^ basic fibroblast growth factor, and hepatocyte growth factor, ^[37]^ and they may act in concert to promote angiogenesis. To evaluate its role in this process, we depleted Ang2 from the postoperative plasmas and checked the effect of the resultant plasmas. We found the treatment did make the plasmas less proangiogenic, suggesting that Ang2 contributes to the proangiogenic property of the plasmas. The postoperative plasma functional data, together with the plasma Ang2 dynamics, support anti-Ang2 therapy for NSCLC in postoperative adjuvant setting.

We acknowledge the limitations of our study. First, we got all the preoperative, POD1 and POD3 blood samples from 154 hospitalized stage I–IIIA patients, but only 2 late samples from each patient for the POD14, POD28, POD42, or POD56 time points because of the sample availability. Samples for the last 4 groups were collected within 3 to 5 days around the indicated dates. The bundling of the data to enable the statistical analysis makes the postoperative Ang2 value at a specific date less accurate. Second, we enrolled patients undergoing standard open thoracic surgery, including wedge resection, segmental resection, sleeve resection, lobectomy, or pneumonectomy. We did not consider the influence of resection types on postoperative plasma Ang2 level because that all the resections are open approaches, with similar incision length and wound area, and that the numbers of different resections vary widely. Even though this analysis is acceptable,[ ^9^ ^38^] caution should be used to interpret the results. Third, we were unable to collect the postoperative plasmas from stage IV patients because of the very limited number of surgeries. Even though preoperative Ang2 level at stage IV is higher than those at the other stages, stage IV postoperative Ang2 might be further elevated and stimulates the preexisting metastases. Surgery for stage IV NSCLC patients is not recommended, which is in line with the results from this study.
